# Genome-scale model of *Pseudomonas aeruginosa* metabolism unveils virulence and drug potentiation

**DOI:** 10.1038/s42003-023-04540-8

**Published:** 2023-02-10

**Authors:** Sanjeev Dahal, Alina Renz, Andreas Dräger, Laurence Yang

**Affiliations:** 1 Department of Chemical Engineering, Queen’s University, Kingston, Canada; 2 Department of Computer Science, University of Tübingen, 72076 Tübingen, Germany; 3 Computational Systems Biology of Infection and Antimicrobial-Resistant Pathogens, Institute for Bioinformatics and Medical Informatics (IBMI), University of Tübingen, 72076 Tübingen, Germany

**Keywords:** Computer modelling, Infection

## Abstract

*Pseudomonas aeruginosa* is one of the leading causes of hospital-acquired infections. To decipher the metabolic mechanisms associated with virulence and antibiotic resistance, we have developed an updated genome-scale model (GEM) of *P. aeruginosa*. The model (*i*SD1509) is an extensively curated, three-compartment, and mass-and-charge balanced BiGG model containing 1509 genes, the largest gene content for any *P. aeruginosa* GEM to date. It is the most accurate with prediction accuracies as high as 92.4% (gene essentiality) and 93.5% (substrate utilization). In *i*SD1509, we newly added a recently discovered pathway for ubiquinone-9 biosynthesis which is required for anaerobic growth. We used a modified *i*SD1509 to demonstrate the role of virulence factor (phenazines) in the pathogen survival within biofilm/oxygen-limited condition. Further, the model can mechanistically explain the overproduction of a drug susceptibility biomarker in the *P. aeruginosa* mutants. Finally, we use *i*SD1509 to demonstrate the drug potentiation by metabolite supplementation, and elucidate the mechanisms behind the phenotype, which agree with experimental results.

## Abstract (web summary)

An updated genome-scale model of *Pseudomonas aeruginosa* explains the metabolic pathways leading to drug resistance and provides a computational platform to design experiments targeting *P. aeruginosa* metabolism.

## Introduction

*Pseudomonas aeruginosa* is a Gram-negative proteobacterium that is metabolically versatile and an opportunistic human pathogen. It is a leading cause of nosocomial infections^1,2^. One of the well-known *P. aeruginosa* infection sites is in the lungs of cystic fibrosis (CF) patients. Such infections can lead to high morbidity and mortality^2^. The ability to resist multiple drugs (including aminoglycosides, quinolones, and *β*-lactams), synthesize virulence factors (e.g., phenazines, proteases, lysins, exotoxins, etc.), and produce biofilms^1–3^ allows *P. aeruginosa* to infect and colonize its host. The pathogenicity of *P. aeruginosa* and the host response can differ between strains^1^.

Genome-scale metabolic models (GEMs) provide a reliable tool for systems study of bacteria^4–6^. GEMs can be advantageous in investigating pathogens because identifying potential intervention strategies can be challenging due to the wide range of genetic mutations and metabolic targets, and niche-specific alteration of metabolic processes^7,8^. GEMs have been utilized for systems investigation of pathogenic species such as *Acinetobacter baumannii*^9,10^, *Klebsiella pneumoniae*^11^, *Mycobacterium tuberculosis*^12^, *Vibrio vulnificus*^13^, and *P. aeruginosa*^14,15^. For the development of GEMs, first a reconstruction of the metabolic pathways of the organism of interest is required, which can then be converted to a mathematical format that can be analyzed using constraint-based modeling and flux balance analysis (FBA) approaches.

Biological functions associated with virulence and drug resistance in *P. aeruginosa* continue to be discovered. Mechanistic models need to be updated continually to incorporate this new knowledge and to facilitate quantitative simulations. For instance, the characterization of multiple terminal oxidases^16–19^ and phenazine-dependent redox reactions continues to expand, and were not included in previous reconstructions^20–22^.

In this study, we developed a highly curated reconstruction of *P. aeruginosa* using PA14 as the strain of choice. It is a hypervirulent strain and belongs to the most common clonal group^23^. A study in 2004 first published its genome, which is highly similar to *P. aeruginosa* PAO1’s genome, but carries two additional pathogenicity islands that contribute significantly to the virulence of PA14^24,25^. The metabolic model (*i*SD1509) is developed using human-interpretable BiGG (Biochemical Genetic and Genomic)^26^ identifiers cross-referencing other databases. The *i*SD1509 contains the largest gene content of any GEM of *P. aeruginosa* to date, and demonstrates high prediction accuracy. Using *i*SD1509, we analyzed four different metabolic traits related to pathogenic behavior and drug resistance. First, using *i*SD1509, we investigated a recently observed pathway for ubiquinone-9 (UQ9) production that is necessary for growth in anaerobic conditions^27^. Since the ability to survive in anaerobic condition is associated with pathogenicity and biofilm growth, we decided to study these phenomena in *Pseudomonas*^28,29^. Hence, the knowledge from literature^27,30^ was incorporated into the current model. Second, phenazine production is associated with *Pseudomonas* survival in biofilm environment and virulence ultimately leading to drug resistance^19,29^. We used the limed-FBA approach^31^ to extend *i*SD1509 to investigate the effect of phenazine biosynthesis on the biomass production at various levels of oxygen availability. Third, since one of the biomarkers of virulence was determined to be gluconate production^32^, we used the model to demonstrate its predictive ability by simulating gluconate production in *Pseudomonas aeruginosa* mutants to provide mechanistic explanation for this metabolic behavior. Finally, through flux sampling analysis using *i*SD1509, we mechanistically elucidate the role of metabolite supplementation in antibiotic susceptibility potentiation to recapture the experimental findings^33^.

## Results

### Model reconstruction and validation

**Fig. 1:**
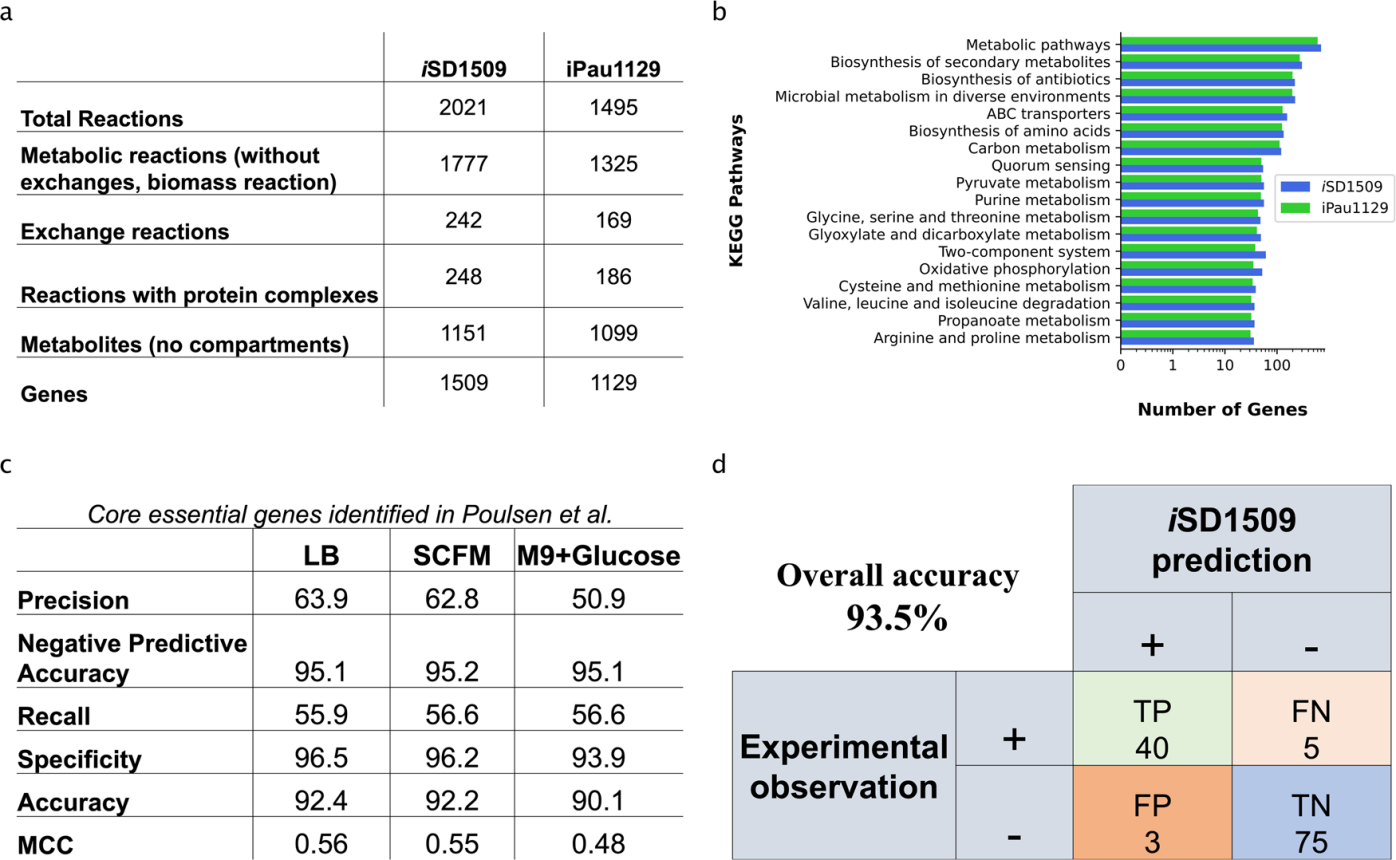
Genome-scale reconstruction of *Pseudomonas aeruginosa* PA14, *i*SD1509 and its predictive assessment. **a** When compared with iPau1129, the latest model *i*SD1509 has considerable increase in the reaction, gene, and metabolite content. Please note that *i*SD1509 contains two biomass reactions: aerobic and anaerobic (thiamine diphosphate (THMPP) removed as one of the constituents). **b** The top KEGG pathways between both reconstructions were compared, and *i*SD1509 evidently has higher gene content in almost every top KEGG pathway. **c** *i*SD1509 was then used for predicting core essential genes determined in a separate study^39^. The model prediction accuracy was found to be >90% in three different media conditions indicating a highly predictive model. **d** Using *i*SD1509, carbon substrate utilization was predicted for a new set of data^40^. The prediction accuracy was computed to be 93.5%.

The reconstruction of *i*SD1509 was developed using the pipeline outlined (Supplementary Fig. 1 and Supplementary Note 1 and 2). We compared the reconstructions of *i*SD1509 and of the previous model iPau1129^14^. The *i*SD1509 model contains considerably higher number of genes, reactions, and metabolites than iPau1129 (Fig. 1a). It possesses 424 more unique genes than in iPau1129. When we compared the top KEGG pathways between the two reconstructions, the number of genes in almost every pathway was higher in *i*SD1509 than in iPau1129 (Fig. 1b). Gene enrichment analyses of *i*SD1509 in both KEGG^34^ and COG^35^ categories are shown in Supplementary Fig. 6. The current MEMOTE^36^ (v. 0.12.0) score of *i*SD1509 is 88% (Supplementary Fig. 2 and Supplementary Data 6).

Next, we validated *i*SD1509 by performing two tests: (1) substrate utilization in minimal media, and (2) gene essentiality. We used the same datasets utilized for validating iPau1129^14^. The *i*SD1509 model can simulate growth on LB, SCFM (Synthetic Cystic Fibrosis Medium), and minimal media containing individual substrates (87 compounds). On the minimal medium, the model predicts substrate utilization with an accuracy of 94.3%. This is a significant improvement compared to the iPau1129 model (80.5%). The predicted biomass yield on the glucose minimal medium (0.089 gDW.(mmol Glc)^−1^) is within the range of experimentally determined yields for *P. aeruginosa* (0.094, 0.085, and 0.118 gDW.(mmol Glc)^−1^) at temperatures of 30 °C, 38 °C, and 41 °C, respectively) under similar conditions^37^.

Next, we compared the metabolic flux analysis (MFA) results with the predicted FBA values for growth on glucose minimal medium. Due to the unavailability of MFA data for PA14 and since both PA14 and PAO1 strains are highly similar^24^, we used the data for PAO1^38^. For the simulation, the minimal medium as described in the study^38^ was used with glucose flux being constrained at 9.7 mmol gDW^−1^ h^−1^. FBA predictions and MFA results were highly correlated (Pearson correlation coefficient: 0.91, *p*-value < 0.001; Supplementary Fig. 3).

For gene essentiality validation, we performed simulations in LB medium. For the genes common between *i*SD1509 and iPau1129 (1,085 genes), *i*SD1509 retained a similar accuracy (*i*SD1509: 90.6% vs. iPau1129: 91.1%) by achieving a >5% higher recall (Supplementary Fig. 4A). The Matthews Correlation Coefficient (MCC) for *i*SD1509 and iPau1129 were 0.49 and 0.48, respectively. An increase in 1% accuracy was achieved when all the genes in *i*SD1509 were considered and the MCC was computed to be 0.45 (Supplementary Fig. 4B).

### Predictive assessment of *i*SD1509

Next, we assessed the model using new datasets. For the gene essentiality data, 321 core essential genes identified in a recent transposon insertion sequence study were used^39^. In the study, the core essential genes were identified in five different media conditions. Because of the availability of three of the five media for in silico simulations – LB, SCFM and glucose minimal (M9), we computed gene essentiality on these three conditions. For LB, SCFM, and M9 minimal medium, overall accuracies were 92.4%, 92.3%, and 90.2%, respectively. Precision ranged between 51.2% (M9) and 64.0% (LB), and recall ranged from 57.2% (LB) to 57.9% (M9 and SFCM) (Fig. 1c). Our model also demonstrated higher prediction accuracy (91.8%, MCC: 0.60) than iPau1129 (91.2%, MCC: 0.55) in LB medium for the core essential genes identified in the study^39^ for the shared genes (1085 genes) between the two models.

We assessed substrate utilization using the data published in Dunphy et al.^40^. The model was simulated on 123 minimal media containing different substrates, and model simulated growth on 43 conditions (model accuracy: 93.5%; Fig. 1d).

### Anaerobic growth

*P. aeruginosa* can utilize nitrate as terminal acceptors for growth in the absence of oxygen. When we first simulated the model in anaerobic condition, it could not simulate growth. We performed similar simulation using the previous model (iPau1129)^14^, and it could not simulate growth either. Two biomass constituents– ubiquinone-9 (UQ9) and thiamine diphosphate (THMPP) were not produced during anaerobic simulation. Since the biomass constituents were predetermined in the previous study^14^, model simulations demonstrated a knowledge gap that needed to be filled for the model to grow anaerobically. For anaerobic THMPP production, no alternative pathway could be determined in *P. aeruginosa* either through exhaustive search in the literature or by annotation-based methods. The bottleneck for THMPP production is iminoglycine formation. In aerobic conditions, glycine oxidase is utilized by *P. aeruginosa*. However, for anaerobic conditions, no alternative source could be identified. One probability is that the enzyme 2-iminoacetate synthase has not yet been identified in this organism. Nevertheless, the possibility of the presence of yet uncharacterized pathways in the organism cannot be ruled out. Hence, we removed this metabolite and adjusted the biomass reaction creating an anaerobic biomass reaction (Supplementary Table 3).

**Fig. 2:**
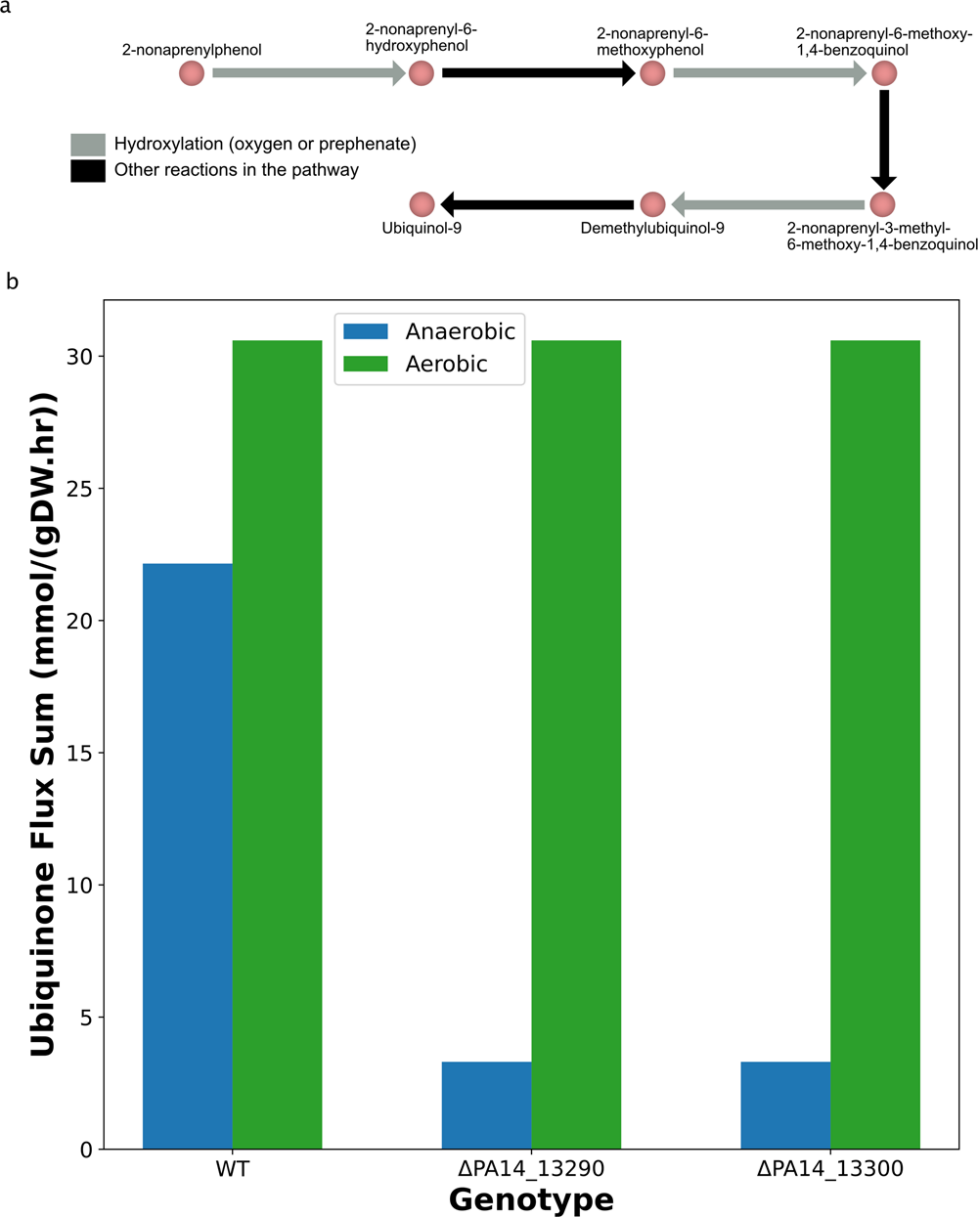
Our model predicts anaerobic growth of *P. aeruginosa* along with higher production of ubiquinone in anaerobic condition than in aerobic one. **a** The proposed pathway for ubiquinone production in both aerobic and anaerobic conditions share same enzymes except for the hydroxylation reactions^27,30^. **b** The model predicts that ubiquinone (UQ9) production is reduced in knockout mutants of the alternative pathway proposed in Fig. 2a in anaerobic condition using nitrate as the terminal electron acceptor (blue bars). However, in aerobic condition (green bars), the production rate is not affected in the mutants. This observation has been demonstrated in the experimental data^27^.

In the case of anaerobic UQ9 production, a recent study^27^ demonstrated that UQ9 can be produced by an alternate pathway within the UQ9 biosynthesis chain. The study demonstrated that biosynthetic proteins are shared between aerobic and anaerobic pathways except for the ones that catalyze the three reactions associated with hydroxylation of intermediate metabolites (Fig. 2a). In an anaerobic environment, an alternative oxidizing molecule (proposed to be prephenate) could be involved^30^. Therefore, we added prephenate-based hypothetical reactions as anaerobic alternatives to the aforementioned three hydroxylation reactions. These reactions are mass-and-charge balanced, and their ΔG^’m^s were computed using eQuilibrator (v. 0.4.1)^41^ to determine their feasibility.

Following the changes, we could simulate anaerobic growth in LB medium. The oxygen or nitrate uptake rates were derived using the model simulations by constraining the growth rates estimated from the experimental data^27^. The flux-sum was computed for UQ9 in aerobic and anaerobic conditions (as described in the Methods section). The model predicted that the UQ9 production is consistent in wildtype and mutants in aerobic conditions whereas it is drastically lowered from wildtype to mutants during anaerobic conditions (Fig. 2b). The predicted result agreed with the observation from a recent experimental study^27^. Additionally, Vo et al.^27^ show that the cellular yield of UQ9 (pmol of UQ9 per mg cell) is higher in anaerobic over aerobic conditions. Cellular yield would be predicted as intracellular concentration (pmol/L) over biomass concentration (gDW/L). However, genome-scale metabolic models do not predict intracellular concentrations^4^. Here, we approximated cellular yield by computing UQ9 turnover as flux sum over growth rate. The computed UQ9 turnover values using *i*SD1509 were 25.2 mmol/gDW (anaerobic) vs. 24.3 mmol/gDW (aerobic). The higher UQ9 turnover in anaerobic vs. aerobic conditions is consistent with the observed differences in UQ9 yield. However, as noted, since intracellular concentrations are not computed, further investigation is needed in future studies.

### Production of pyocyanin in *P. aeruginosa* can be simulated by extended *i*SD1509

We next used *i*SD1509 to investigate phenazine production in *P. aeruginosa*. *Pseudomonas* can produce and secrete phenazines (e.g., pyocyanin), which possess redox properties and can cycle in and out of the cell. Usually, they are reduced within the cell, then they go to the extracellular space to get oxidized (by donating electrons to acceptors such as oxygen), and finally get transported back in the cell to complete a redox cycle^20,42^. This knowledge has been added to *i*SD1509 using the information derived from the literature review^21,43–48^ to use the model for the identification of potential intervention strategies that target phenazine redox cycle (Supplementary Fig. 5A).

Phenazine production has been shown to be stimulated by low oxygen tension^49^. Further, phenazines are involved in the survival of *P. aeruginosa* in biofilm environment in which oxygen is limiting^20,42^. The chronic stage of CF infections, in which *P. aeruginosa* is fully adapted, is characterized by formation of biofilms^50^. Phenazines have been detected in samples associated with CF infections in various studies^51,52^. Therefore, phenazines have been proposed to be important molecules for the survival during CF infections^53^.

To simulate phenazine production, since normal FBA models (*i*SD1509) do not dilute metabolites that are not part of the biomass reaction, we applied limed-FBA on *i*SD1509. As phenazines take part in active reactions^31^ in low-oxygen condition, they need to be synthesized as cell divides. With limed-FBA, phenazines are diluted and replenished to achieve mass-balance^31^ (Supplementary Fig. 5B). Using this framework, we studied the effect of oxygen availability and phenazine production on the growth rate of *P. aeruginosa* (Supplementary Fig. 5B, C). For the simulation, we used LB medium with oxygen import flux (from extracellular to periplasm) being constrained over a range to simulate different oxygen availabilities. The range of flux was estimated using sensitivity analysis (Supplementary Methods). We identified that pyocyanin production was not sensitive to oxygen uptake rates for the flux range that we tested (0.01 mmol gDW^−1^ h^−1^ to 0.5 mmol gDW^−1^ h^−1^) as the pyocyanin synthesis flux decreased from 0.000904 mmol gDW^−1^ h^−1^ to 0.000882 mmol gDW^−1^ h^−1^, respectively, changing by only 2.5%. Hence, we decided to use 0.5 mmol gDW^−1^ h^−1^ as the lower threshold for oxygen uptake rate. Using the sensitivity analysis, we also decided to use 10 mmol gDW^−1^ h^−1^ as the upper threshold for oxygen uptake rate as the pyocyanin production rate decreased by half from the maximum production value (Supplementary Fig. 5D).

**Fig. 3:**
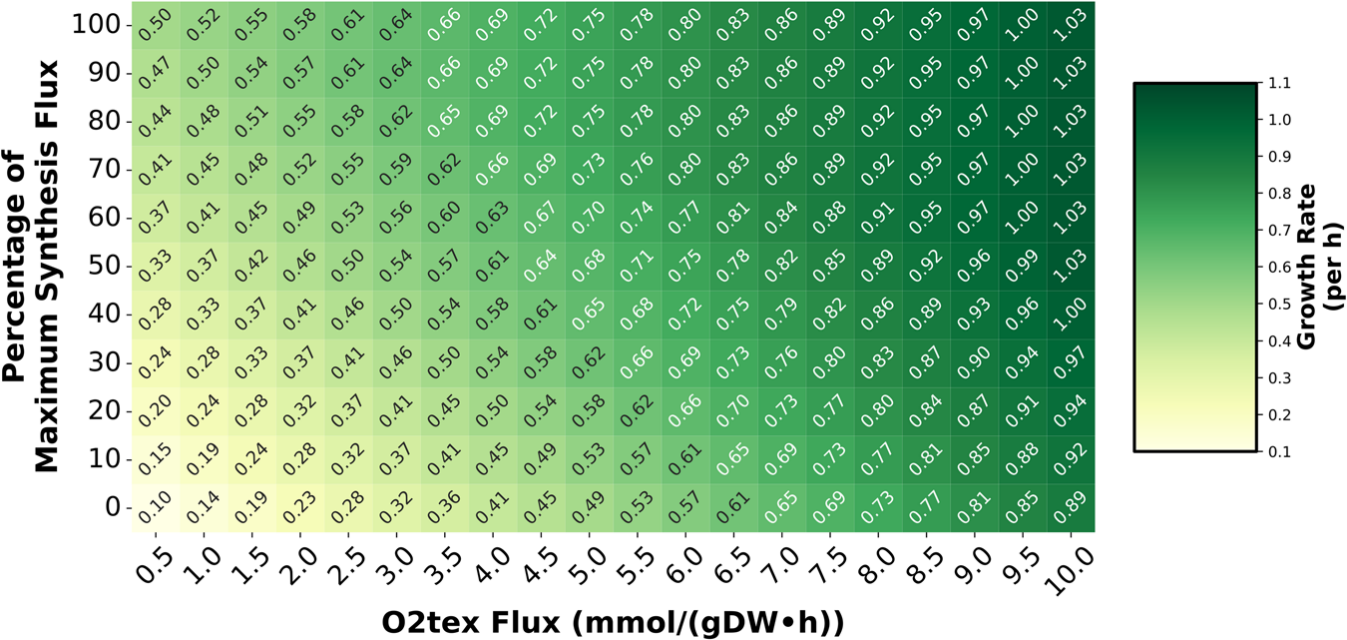
The limed-FBA model can predict the biomass production dependence on pyocyanin biosynthesis and oxygen availability. Simulations were performed over a wide range of oxygen uptake (from extracellular to periplasm) and pyocyanin biosynthesis flux constraints (relative scale shown as percentage such that 100% refers to the highest flux computed at 0.5 mmol gDW^−1^ h^−1^ oxygen import flux) by optimizing for biomass production. Each cell refers to the growth rate computed by simulating the model at fixed oxygen uptake (reaction: O2tex) rate and pyocyanin synthesis flux. The color scheme represents increasing growth rates from light yellow to dark green. For oxygen-limited condition (lower oxygen import flux), the effect of pyocyanin production on the growth rate is more profound compared to that in more oxygen-rich condition (higher oxygen import flux).

For each of those flux constraints, pyocyanin synthesis flux was also constrained over a range (0 mmol gDW^−1^ h^−1^ to maximum value (0.000882 mmol gDW^−1^ h^−1^ computed for 0.5 mmol gDW^−1^ h^−1^ oxygen import flux)). For each value of oxygen import flux and pyocyanin biosynthesis flux, the growth rate was computed. With this analysis, we observed that for lower oxygen import flux (less oxygen available to the cell), the effect of pyocyanin production on the growth rate is pronounced. In contrast, when more oxygen is available to the cell, pyocyanin synthesis does not considerably contribute to the biomass production (Fig. 3 and Supplementary Fig. 5C). For instance, at lowest oxygen uptake flux (0.5 mmol gDW^−1^ h^−1^), the increase in growth rate over the pyocyanin production flux range is 0.41 h^−1^ whereas at highest oxygen uptake rate (10 mmol gDW^−1^ h^−1^), the increase in growth rate is 0.14 h^−1^.

As growth rate is proportional to pyocyanin production flux at lower oxygen availability, the *i*SD1509_limed mechanistically demonstrates that at such conditions, *Pseudomonas* is forced to divert resources towards phenazine production to counteract the imbalance in the intracellular redox state caused by the reduced availability of oxygen^20^. Therefore, at lower oxygen availability (e.g., within biofilms, chronic CF infections), the model predicts that phenazines are required for biomass production (Fig. 3).

### Identification of potential mutants that overproduce gluconate

The loss of function mutation in *rpoN* is common among the clinical isolates in CF patients^54,55^. The *rpoN* mutant is a significant gluconate producer. Behrends et al. proposed that gluconate production is positively (weak but significant) correlated to reduced antibiotic susceptibility. Further, the investigators demonstrated that only the mutant of one of the indirect targets of RpoN, 6-phosphogluconate dehydratase (6PGDH, PA14_22910) could replicate the gluconate overproduction phenotype of the *rpoN* mutant^32^.

**Fig. 4:**
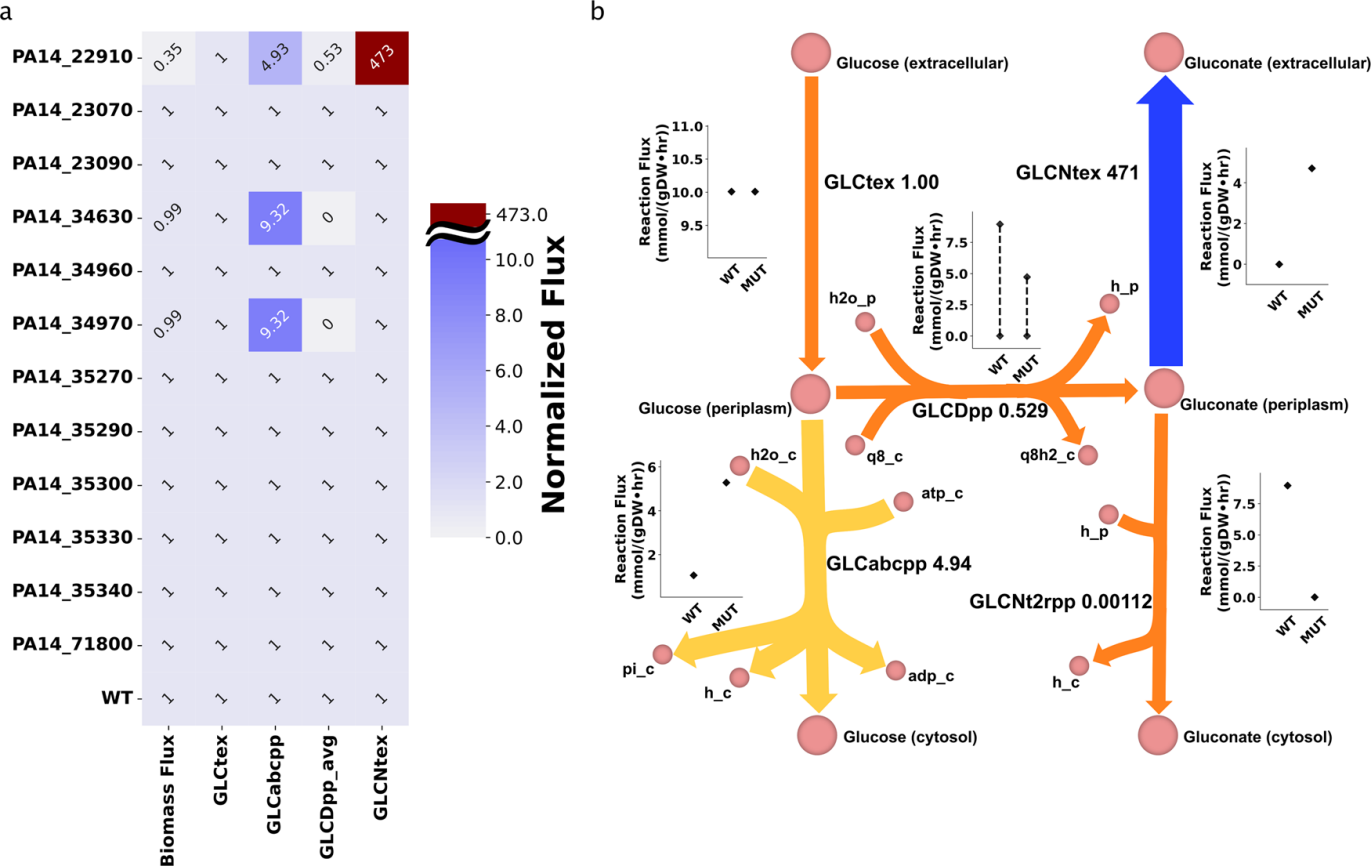
Our model can accurately predict gluconate production in the mutants of the genes regulated by RpoN. Twelve genes regulated by RpoN were in silico knocked out in this study in order to recapitulate the results of experimental study^32^. **a** FBA simulations were able to accurately predict that only *edd* mutant (PA14_22910) produces considerable amount of gluconate as shown by higher flux in reaction GLCNtex (demonstrated in Fig. 4b) compared to the wildtype. Furthermore, the decrease in the growth rate of the mutant was also recapitulated in this study (left). For calculations, all flux values were increased by 0.01 for normalization purpose. The fluxes of the reactions catalyzed by glucose dehydrogenase (GLCDpp and GLCDpp_q9) have been averaged (column GLCDpp_avg). Then, mutant reaction fluxes were divided by respective wildtype reaction fluxes. **b** To further characterize the *edd* mutant, FVA simulations were carried out in order to examine the flux range of desired reactions in both wildtype and mutant. Gluconate excretion is required by the mutant for optimal growth in the given condition. Furthermore, the glucose flux in the mutant is divided between glucose transport and glucose dehydrogenase reactions whereas in the wildtype, majority of the flux is channeled towards the Entner-Doudoroff pathway through glucose dehydrogenase reaction (Supplementary Fig. 3). The values (after the reaction names) are derived by dividing the average reaction fluxes of the *edd* mutant by those of the wildtype. To avoid division by zeroes, all the simulation values were first increased by 0.01. Absolute values are reported in the figure. In the figures: Biomass biomass reaction, GLCtex glucose transport (from extracellular to periplasm), GLCabcpp glucose transport (from periplasm to cytosol), GLCDpp/GLCDpp_avg glucose dehydrogenase, GLCNt2rpp gluconate transport (from periplasm to cytosol).

We used *i*SD1509 to recapitulate the results of the study^32^ on the glucose minimal (M9) medium. Of the knockouts of the twelve indirect targets of RpoN, the FBA simulations accurately predicted that only the deletion of 6PGDH (*edd* gene, locus tag: (PA14_22910)) leads to a significant increase in the gluconate production compared to the wildtype (Fig. 4a). The model also correctly predicted that the growth of the *edd* mutant is considerably affected as normalized gluconate secretion flux is more than 470 times higher in the mutant than that in the wild-type. The simulations provided insights into other mutants such as deletion of gluconate symporter gene (PA14_34630) leading to increased flux through glucose transport reaction (GLCabcpp, from periplasm to cytosol; Fig. 4a).

To further expand on the previous findings by Behrends et al., and to mechanistically explain the gluconate overproduction phenotype, we performed FVA simulations (by fixing growth rate at 100% of its maximum value). We proposed that the gluconate production in *edd* mutant was a requirement for the optimal growth. The gluconate secretion (GLCNtex) reaction flux range (absolute value) in the mutant (4.71 mmol gDW^−1^ h^−1^) is fixed at a higher rate than in the wildtype (0 mmol gDW^−1^ h^−1^) suggesting that for the optimal growth of the mutant, gluconate is forced to be transported out of the cell (Fig. 4b). Thus, gluconate overproduction is explained by mass balance, thermodynamics that constrains reaction reversibility, and growth optimality.

### Fumarate supplementation leads to an increased TCA cycle and proton motive force activities

Meylan et al.^33^ showed that the addition of fumarate along with tobramycin leads to greater drug uptake and activity whereas glyoxylate protects the cells from tobramycin lethality. The study showed that the higher tobramycin uptake and activity in fumarate-containing medium is due to greater proton motive force (PMF) and increased respiration rate caused by higher flux through the TCA cycle, respectively. Likewise, in glyoxylate-containing medium, glyoxylate, by directly inhibiting *α*-ketoglutarate dehydrogenase, diverts the flux away from TCA cycle towards the glyoxylate shunt leading to reduced TCA cycle activity. We used *i*SD1509 to test an alternative, i.e., whether the law of optimal growth can explain drug protection by glyoxylate supplementation. Furthermore, we used *i*SD1509 to study the pathway utilization differences between the two metabolite supplementations.

**Fig. 5:**
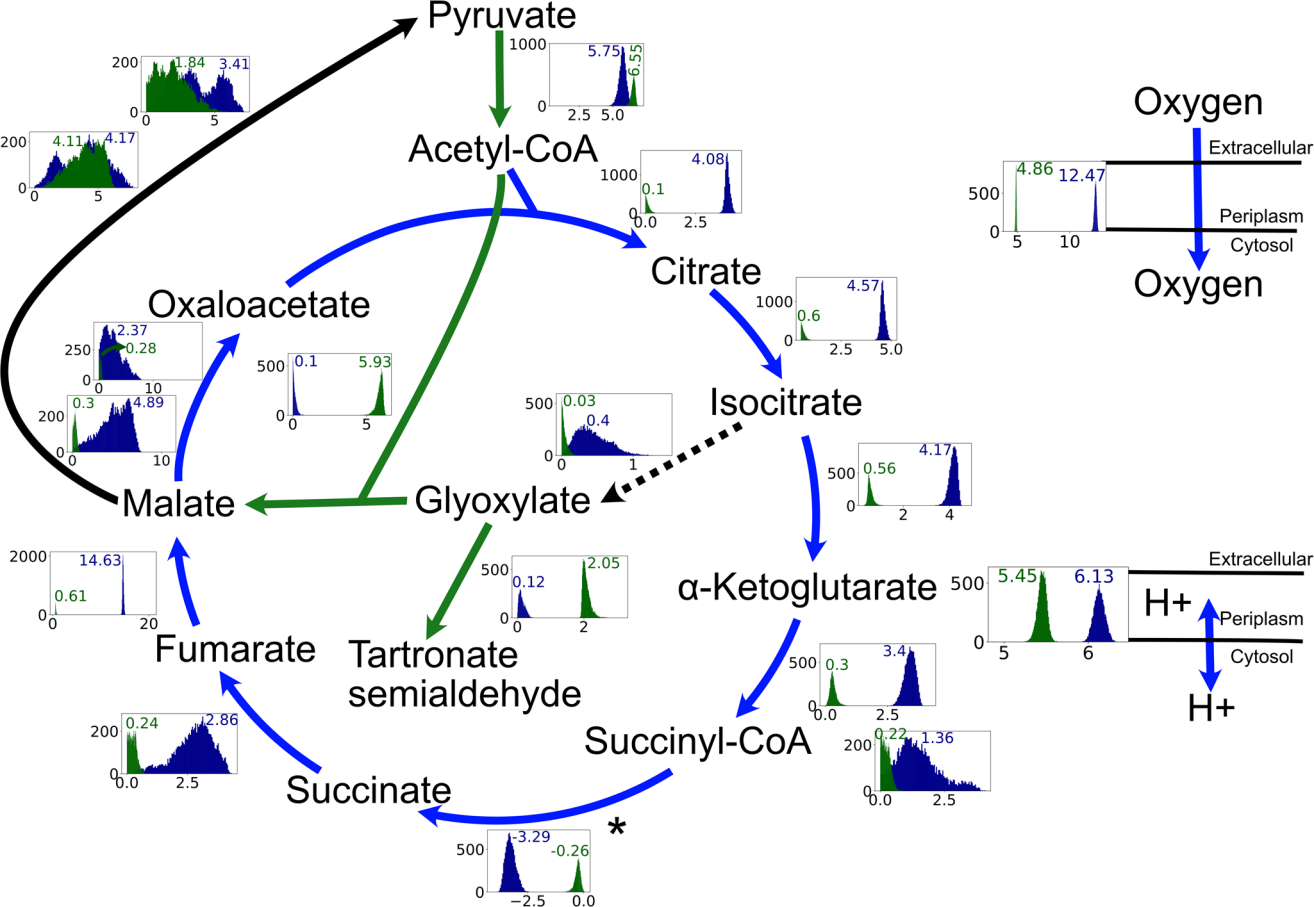
The *i*SD1509 model predicts that the fumarate supplementation causes increased PMF activity and higher flux through TCA cycle leading to greater oxygen uptake rate. Flux sampling analysis (*n* = 10,000 samplings) was performed in minimal medium containing fumarate or glyoxylate to identify the flux distributions of TCA cycle reactions. According to the analysis, the TCA cycle is significantly upregulated in fumarate-supplemented medium compared to the glyoxylate-supplemented one. The asterisk for reversible succinyl-CoA synthetase reaction is to note that the reaction flux is negative. In glyoxylate-supplemented medium, the flux from acetyl-CoA is shunted towards the glyoxylate cycle as shown in the figure. The flux goes through the reactions catalyzed by malic enzymes to regenerate the cofactors – NADH and NADPH. This is observed along with a lower PMF activity, reduced TCA cycle flux, and decreased oxygen uptake rate as compared to fumarate-supplemented medium. All the sampling results (boxes with flux distributions where green: glyoxylate and blue: fumarate) are for reaction fluxes except for proton for which flux-sum yield was computed from the sampling data. Except for the reaction ME2 (pyruvate to malate), the differences in fluxes for all reactions shown are statistically significant. Arrow colors indicate median fluxes being higher in one condition versus the other such that green: glyoxylate, blue: fumarate, black and bold: similar, and black and dotted: low flux in both conditions.

We simulated *i*SD1509 on minimal (M9) medium containing glyoxylate or fumarate, and low amounts of citrate by optimizing for the biomass production. The medium was derived from the study^39^ and the flux constraints were chosen based on a separate minimal medium information provided by Papin Lab. Citrate flux was arbitrarily constrained at low flux at 0.5 mmol gDW^−1^ h^−1^. Glyoxylate was not predicted to be a growth-inducing substrate in the substrate utilization assessment step. However, Meylan et al.^33^ performed wet lab experiments clearly showing that glyoxylate supplementation in culture media induced phenotypic changes to *P. aeruginosa*, including suppression of electron transport chain activity. Such phenotypic responses likely require uptake of glyoxylate into the cell, presumably via an uncharacterized transporter (personal communication). Therefore, an artificial glyoxylate uptake reaction was added to the model. We performed a flux sampling analysis by constraining the biomass flux to 90% of the estimated growth rate (by FBA) on both media assuming the cells optimize for high growth rates to counterbalance the effect of the drug killing. For proton flux calculations, we computed the flux-sum yield of periplasmic proton (as described in the Methods). Then, we compared the median fluxes of the reactions pertaining to TCA cycle and glyoxylate cycle between the two media conditions. The model predictions agreed with Meylan et al.^33^ that the glyoxylate shunt indeed drives the flux away from TCA cycle in glyoxylate-containing medium. Likewise, the simulations also indicate that the glyoxylate flux is diverted towards the reactions catalyzed by malic enzymes, which recycle the necessary cofactors– NADH and NADPH. Unlike the Meylan study, the model predicted higher flux activity through pyruvate dehydrogenase reaction in the glyoxylate minimal medium. Moreover, the production of oxalate and glycolate were not confirmed by the model predictions. Instead, the glyoxylate flux was diverted towards the reaction catalyzed by glyoxylate carboligase. We also observed that the oxygen uptake rate and proton flux-sum yield were higher in the fumarate- than in the glyoxylate-supplemented medium, which leads to increased drug uptake and activity in fumarate treatment^33^ (Fig. 5). Therefore, using our analysis, we have demonstrated that the law of optimality could also explain drug potentiation and drug protection by fumarate and glyoxylate supplementation, respectively.

## Discussion

*Pseudomonas aeruginosa* is an extensively studied organism in association with human infections especially in CF lungs. Since it is known to possess multiple mechanisms of drug resistance (e.g., lipopolysaccharide modification, overexpression of efflux pumps)^3^ and an ability to survive within the biofilm environment, eradication of *Pseudomonas* infections can be challenging. Since it contains extensively curated literature-derived knowledge, *i*SD1509 is highly predictive and can be used as a platform to design experiments and strategies to combat infections caused by *P. aeruginosa*.

In this study, we have demonstrated that *i*SD1509 is a highly predictive and reliable model with accuracies of 90.2–93.4% and 93.5% for gene essentiality and substrate utilization predictions, respectively. Further, guided by the knowledge gap in our model, we identified that the biosynthesis of ubiquinone-9 is crucial for the anaerobic growth of *P. aeruginosa*, and added the information from recent studies^27,30^ to fill this gap allowing model to simulate growth in anaerobic conditions. The model also predicted that the survival of *P. aeruginosa* in low oxygen environment (such as within biofilms produced in chronic CF infections) is possible due to phenazine production and provided mechanistic insight into their biosynthesis. With the model, the role of phenazines in CF condition can be explored further. For instance, how different media condition affect phenazine production can be studied using this model^56^. Furthermore, using *i*SD1509 along with individual-based models, biofilm production and the role of phenazines in CF infections can now be investigated^57^.

The model successfully identified the mutant that produced higher amounts of gluconate than the wildtype among twelve candidates. Using *i*SD1509, more computational experiments can be designed to identify other mutants that overproduce gluconate. Likewise, other possible biomarkers of drug resistance can be predicted. Furthermore, using multi-strain modeling approaches^58^, models of various *P. aeruginosa* isolates can be simulated to compare biomarker production simultaneously. Other host pathoadpative mechanisms such as pyruvate overproduction in pyruvate dehydrogenase mutant strains^32,59^ can also be investigated using *i*SD1509.

Finally, we used *i*SD1509 to recapitulate the potentiation of an antibiotic by metabolite supplementation^33^. The model correctly differentiated between metabolites that increased drug lethality versus those that did not and offered mechanistic explanations for these responses. The model *i*SD1509 predicts that *Pseudomonas* possesses mechanisms to uptake glyoxylate by yet unknown systems as the simulations (after addition of artificial reaction) were comparable to the experimental results. Namely, supplementing glyoxylate diverted flux away from the TCA cycle, lowering the respiration rate that consequently led to lower drug activity. Supplementing the medium with fumarate caused higher oxygen uptake rate due to higher TCA cycle activity which leads to higher drug activity^33^. Likewise, PMF (and ultimately drug uptake) was higher in the fumarate-containing medium than in the glyoxylate-containing medium. These results suggest that our model can be used to design antimicrobial strategies based on metabolic mechanisms, including metabolite supplementation, against *P. aeruginosa*. This genome-scale model can be integrated with machine-learning approaches for mechanistic understanding of antibiotic efficacy. This model can be applied in such pipelines as demonstrated in recent studies^60^.

In conclusion, we have developed a highly curated model that provides a computational platform to design experiments targeting *P. aeruginosa* metabolism at various growth states including within the biofilm environment. Likewise, novel predicted targets of *P. aeruginosa* (i.e., pyocyanin redox cycle) can now be investigated to identify the best strategy to inhibit the growth of the pathogen. Overall, we expect the model to significantly accelerate our understanding of *P. aeruginosa* to combat the associated infections.

## Methods

### Model simulations

For the simulation of the model, we performed flux balance analysis (FBA) on conditions reflecting the media used in a particular experimental study (Supplementary Table 1 and Supplementary Data 5). Gurobi (v. 9.5.1) was used for all model simulations. The flux bounds were derived from previous study^14^ unless stated otherwise. Carbon source uptake rates (for minimal medium) were constrained at 10 mmol gDW^−1^ h^−1^ whereas uptake rates for terminal acceptors (oxygen or nitrate) were constrained at 20 mmol gDW^−1^ h^−1^. Any changes to these bounds are mentioned within the text. For a more detailed method on media formulation, please see Supplementary Methods. The FBA simulations were performed using COBRApy package (v. 0.18.1)^61^. In most simulations excluding anaerobic growth predictions, we used the aerobic biomass reaction as the objective function. For anaerobic growth predictions, we used the anaerobic biomass reaction. If the biomass flux was computed to <10^−5^ h^−1^, “no growth” was assigned.

#### Ubiquinone flux-sum

For these simulations, first the growth rate was determined using *growthrates* package (v. 0.8.4) in R (v. 4.2.0). The function *fit_growthmodel* was applied for logistic growth model with the *BFGS* method for both aerobic and anaerobic conditions. The growth data used for this analysis was estimated from Vo et al.^27^ using Engauge Digitizer (v. 12.1)^62^. The model was constrained using the derived growth rates to determine the uptake fluxes for the oxygen or nitrate in respective conditions. In aerobic condition, oxygen uptake rate was constrained at 20 mmol gDW^−1^ h^−1^ and nitrate uptake rate at zero and vice-versa for anaerobic condition. Likewise, the ubiquinone-8 reactions were turned off for both condition whereas the oxygen-associated terminal oxidases were shut down for anaerobic condition only. Next, the computed uptake rates were added as lower bounds on the model. The gene knockouts were performed using *delete_model_genes* module in COBRApy in aerobic and anaerobic conditions. The simulations were performed by optimizing for biomass production followed by the computation of flux-sum for ubiquinone-9 for wildtype and mutants.

#### Gluconate production

For the computation of gluconate production, both FBA and flux variability analysis (FVA)^63^ (with loopless method and fraction of optimum set to 1) were applied. All simulations were performed in M9 medium. Experimental data to validate gluconate production were obtained from Behrends et al.^32^, where measurements for RpoN mutant and wild-type were performed in M9 media. Gluconate production for mutants besides RpoN were measured by Behrends et al.^32^ in SFCM media. However, for consistency and to facilitate interpretation, we simulated all mutants in M9 medium (a minimal medium) and found that these simulations were consistent with experiments in SFCM medium (a rich medium).

To compare the wild-type and mutant fluxes, we applied a heuristic approach. First, any computed flux values were increased by 0.01 to avoid “division by zero” error as mutant reaction fluxes were divided by those of the wildtype during normalization step. For FBA simulations, the reaction fluxes of the mutant were divided by those of the wildtype. For FVA fluxes, an average flux of each reaction was calculated by taking the mean of minimum and maximum flux values for both mutant and wildtype. Then, the mutant average reaction flux values were normalized by those of the wildtype.

#### Pyocyanin production

For pyocyanin production simulations, a sensitivity analysis (Supplementary Methods) was performed by fixing the oxygen bounds between 0 mmol gDW^−1^ h^−1^ to 20 mmol gDW^−1^ h^−1^ (with step 0.01 mmol gDW^−1^ h^−1^) to compute pyocyanin synthesis flux to determine the range of oxygen uptake rates to use for future simulations (Supplementary Fig. 5D).

For a detailed information about the model constraints and objective function used for the simulations (UQ9 flux-sum, pyocyanin production, gluconate overproduction, metabolite supplementation increasing proton motive force activity), please see Supplementary Methods.

### Addition of uncharacterized reactions

For the addition of uncharacterized reactions derived from the literature, we first checked for mass-and-charge balance. Then, we entered the reactions in the eQuilibrator program (v. 0.4.1)^41^ in Python (v. 3.7.7). The parameters- pH = 7.5, ionic strength = 0.25 M, temperature = 25 °C, control of magnesium ion (pMg) = 3 were used. Physiological concentrations (aqueous reactants at 1 mM) were assumed for the calculation of Gibbs free energy of transformation (ΔG^’m^). Reactions with ΔG^’m^<0 were added to the model. For instance, reactions NADNAQ_q8, NADNAQ_q9, rxn12880_pphn, rxn13107_pphn, rxn13108_pphn were added using this method.

### limed-FBA

For the simulation of dilution of cofactors (e.g., pyocyanin), we used limed-FBA approach^31^. Briefly, all the reactions were first made irreversible. Then, for the reactions consuming or producing the desired cytosolic cofactor (e.g., pyocyanin), a small dilution constant (*ϵ*) was applied such that

1 $$(S-\epsilon {S}^{{{{{{{{\rm{binary}}}}}}}}})\cdot v=0$$

where ${S}_{ij}^{{{{{{{{\rm{binary}}}}}}}}}=1$ if *S*_*ij*_ ≠ 0, and *ϵ*_*ii*_ for metabolite *i* was computed using the following formula,

2 $${\epsilon }_{ii}=\frac{0.1}{1000\cdot {\sum }_{j}{S}_{ij}^{{{{{{{{\rm{binary}}}}}}}}}}$$

To not double-dilute biomass related metabolites, their *ϵ*_*ii*_ was made zero. Likewise, for simple transport reactions in which no chemical transformations (e.g., pyocyanin(extracellular) → pyocyanin (cytosol)) occur, their binary coefficients were assigned zeros to avoid any high-flux loops.

### Superoxide leakage

We reconstructed the superoxide leakage by creating a net reaction composed of two: (1) normal reaction, in which water is produced as a by-product of redox metabolism (0.5 o2_e + pyoh2_e → h2o_e + pyo_e), and (2) leaky reaction in which superoxide is produced as a by-product (2.0 o2_e + pyoh2_e → 2.0 h_e + 2.0 o2s_e + pyo_e). We estimated the ROS leakage stoichiometric coefficient (*κ*) by taking into account the changes in the amount of pyocyanin (which can lead to superoxide production) and hydrogen peroxide (a product of superoxide dismutation) produced after 24 hours of growth^21^. According to this method,

3 $$\kappa =\frac{1}{\frac{\Delta O{D}_{{{{{{{{\rm{phenazine}}}}}}}}}}{\Delta O{D}_{{{{{{{{\rm{hydrogen}}}}}}}}{{{{{{{\rm{peroxide}}}}}}}}}}}$$

The Δ*OD*s represent the ratios of absorbance measurements at 0 and 24 hours for either phenazine or hydrogen peroxide which were approximated from Das et al.^21^. Then, we multiplied the stoichiometric coefficients of reaction (2) by *κ* and those of reaction (1) by 1-*κ* before adding up the reactions to generate a net reaction. The value of *κ* was estimated to be 0.29. Therefore, the net reaction used in the model is: 0.935 o2_e + pyoh2_e → 0.58 h_e + 0.71 h2o_e + 0.58 o2_e + pyo_e

### Flux-sum analysis

For the metabolites of interest, we performed flux-sum analysis^64^. Briefly, flux-sum (Φ_*i*_) for metabolite i can be computed using the following formula,

4 $${\Phi }_{i}=\frac{1}{2}\mathop{\sum}\limits_{j}| {S}_{ij}{v}_{j}|$$

where *j* is the index for a reaction in which the metabolite *i* participates in.

For the computation of flux-sum yield, the flux-sum was divided by the substrate flux multiplied by the number of carbon atoms in the substrate (for proton motive force (PMF)). For computation of UQ9 turnover, the flux-sum was divided by the growth rate.

### Flux sampling

We applied flux sampling approach^65^ in the COBRApy package^61^ by using optGpSampler^66^ with 10,000 samplings in both fumarate and glyoxylate minimal media. The growth rate was constrained to 90% of the rate predicted in FBA simulations in the respective media condition. By using the validate method of the optGpSampler, only valid samples were kept for further analysis. Furthermore, we used the autocorrelation plots and trace plotting for convergence analysis.

### Statistics and reproducibility

To compare the distributions of reaction fluxes or metabolite flux-sums (*n* = 10,000 samplings) between the two different media conditions (fumarate versus glyoxylate), we performed Wilcoxon rank-sum test. In this test, the alternative hypothesis is that measurements in one sample is more likely to be larger than those in the other sample. A *p*-value ≤ 0.05 is considered statistically significant. Data were analyzed using *scipy* package (v. 1.7.3) in Python. Median of the distribution was computed using *numpy* package (v. 1.21.5).

### Visualization of flux data

We first created a preliminary Escher^67^ map in the Python-based framework, and then exported the map to web-based Escher tool for appropriate changes. Default settings were applied except for the customization of color and size of the edges for better visual representation. Then, we modified the Escher maps using vector graphic tool (Affinity Designer, v. 1.9.3 and Inkscape, v. 1.1.2).

We produced the heatmaps using *seaborn* package (v. 0.10.1) in Python. All the respective flux values were divided by their respective wild-type values. To avoid division by zeroes, all computed fluxes were first offset by 0.01. For color range, robust quantile computation function was applied.

### Reporting summary

Further information on research design is available in the Nature Portfolio Reporting Summary linked to this article.

## Supplementary information

Supplementary Information

Description of Additional Supplementary Data

Supplementary Data 1

Supplementary Data 2

Supplementary Data 3

Supplementary Data 4

Supplementary Data 5

Supplementary Data 6

Supplementary Data 7

Reporting Summary

## Data Availability

The model (including report from FROG curation tool^68^) can be accessed through the BioModels database^69^ at https://www.ebi.ac.uk/biomodels/MODEL2205090001. By default, the model is constrained for aerobic growth in LB rich medium. The models used for model curation process in this study are described in Supplementary Table 2. The data used for updating the draft reconstruction are provided in Supplementary Data 1–4. The source data underlying Figs. 2, 3, 4, and 5 are provided as Supplementary Data 7. All other data are available from the corresponding author (or other sources, as applicable) on reasonable request.

## Code availability

Sample codes are available at https://github.com/QCSB/iSD1509M. The version of the repository used for this manuscript has been archived in Zenodo (10.5281/zenodo.7545467)^70^. The code is written in Python (v. 3.7.7). The required packages are – *scipy* (v. 1.7.3), *numpy* (v. 1.21.5), *cobrapy* (v. 0.21.0), *pandas* (v. 1.3.5), *matplotlib* (v. 3.5.1), and *seaborn* (v. 0.10.1).
