# Supplementary material for: Genome-scale model of Pseudomonas aeruginosa metabolism unveils virulence and drug potentiation
Source: Commun Biol. 2023 Feb 10;6:165. doi: 10.1038/s42003-023-04540-8 (PMC9918512; doi:10.1038/s42003-023-04540-8)
Supplement: Supplementary file 1 — Supplementary Information [file 42003_2023_4540_MOESM1_ESM.pdf]

Supplementary Information

Genome-scale model of *Pseudomonas aeruginosa* metabolism unveils virulence and drug potentiation

Sanjeev Dahal[1], Alina Renz[2,3], Andreas D Andreas Dräger[2,3], Laurence Yang[1,*]

[1]Department of Chemical Engineering, Queen's University, Kingston, Canada

[2]Department of Computer Science, University of Tübingen, 72076 Tübingen, Germany

[3]Computational Systems Biology of Infection and Antimicrobial-Resistant Pathogens, Institute for Bioinformatics and Medical Informatics (IBMI), University of Tübingen, 72076 Tübingen, Germany

Correspondence should be directed to L.Y. (laurence.yang@queensu.ca)

Supplementary Note

1. Initial reconstruction and manual refinement

We used CarveMe[1] to create a draft reconstruction that contained 2196 reactions and 1482 genes. Following this, we added the biomass reaction from the previous SEED model (iPau1129[2]). We gap-filled our model in LB rich medium to produce all the biomass constituents by adding the reactions from iPau1129 or from other BiGG models.

Next, we curated our model through a semi-automated approach. We chose seventeen different BiGG models to perform a bidirectional blast hit for each of the reactions present in the draft model (Supplementary Table 2). We then manually checked the reactions for any discrepancy in the gene-protein-reaction (GPR) associations made by CarveMe and by our approach. Any discrepancy was resolved using the knowledge from other databases (e.g., IMG[3], KEGG[4]). Reactions removed from the model are provided (Supplementary Data 1).

Using carbon substrate essentiality[2] and gene essentiality data[5], we iteratively filled more knowledge gaps either through annotation- or literature-derived information. A list of reactions that were added to the model is provided (Supplementary Data 2). We also utilized gene essentiality data to fix GPR associations. Likewise, biomass reaction was also modified during this process to better reflect the gene essentiality data.

We next added any extra genes in iPau1129 not present in our model. This process led to the addition of reactions related to alginate production, rhamnolipid production, pyochelin production, etc. Only 44 genes from iPau1129 are missing in the current model (Supplementary Data 3). Finally, we checked and corrected any mass-and-charge imbalance, first by applying the knowledge from well-curated BiGG[6] models (*i*JN1462[7] and *i*ML1515[8]), which led to only 129

metabolites that required additional manual curation. The formula and charges of those metabolites were changed either using information from metabolite databases (BiGG[6], MetaNetX[9], PubChem[10] or ModelSEED[11]) or manually by mass-and-charge balancing the reactions (Supplementary Data 4).

2. Literature derived curation and addition of reactions

We curated the knowledge derived from literature to add to the reconstruction to make the model more strain-specific. The reactions pertaining to multiple terminal oxidases, reactive oxygen species elimination, n-alkane degradation, phenazine-associated metabolism, oxalate production, rubredoxin-based metabolism and H+-translocating NADH:quinone oxidoreductases were added. This led to the development of a model possessing 1509 genes. The model (referred to as *i*SD1509) contains 2023 total reactions and 1151 unique metabolites and possesses three compartments.

We analyzed the additional genes in *i*SD1509 by enrichment in both KEGG[4] and COG[12] categories (Supplementary Fig. 6). In KEGG, notable enriched pathways were oxidative phosphorylation, sulfur metabolism, folate biosynthesis, glyoxylate and dicarboxylate metabolism, and biofilm formation. In COG, the top three categories were energy production and conversion (C), amino acid transport and metabolism (E), and inorganic ion transport and metabolism (P). We also examined the top 20 KEGG pathways of the new metabolic reconstruction (Supplementary Fig. 6C). The greatest number of genes in *i*SD1509 belong to the category “Metabolic pathways.” Interestingly, other significant pathways are associated with biosynthesis of secondary metabolites, metabolism in diverse environments, biosynthesis of

antibiotics, indicating a reconstruction of a metabolically versatile organism (Supplementary Fig. 6C).

Supplementary Methods

1. Initial Reconstruction

For the initial reconstruction, CarveMe[1] with gap-filling function in LB medium was utilized. From the previous *P. aeruginosa* model (iPau1129)[2], we added the biomass reaction along with other required reactions to simulate the growth of the model on LB medium. For this process, we first converted the ModelSEED[11] metabolite identifiers of iPau1129 to BiGG identifiers using custom mapping database. Furthermore, the bounds of non-growth associated reaction (NGAM) were also added from iPau1129. Then, we removed all the artificial sink and demand reactions added by CarveMe by making sure that the respective metabolites can be produced or consumed by added reactions which are supported by gene evidence.

Then, the *Pseudomonas aeruginosa* PA14 reactome was inspected for the gene-protein-reaction (GPR) associations using a custom pipeline (Supplementary Fig. 1). First, seventeen models from BiGG database and their respective protein FASTA files were downloaded (Supplementary Table 2). Then, we performed a bidirectional blast hit (BBH) for all the reactions present in the model by prioritizing the strains that are taxonomically closer to PA14 strain. We applied a stringent method such that only the top hits in both directions were considered as the correct genes. If not, we manually inspected the top blast hits in different databases including IMG[3] and KEGG[4]. These GPR associations were then used as alternatives to

CarveMe predictions. We performed a rigorous manual check for the reactions whose GPR associations were derived from the BBH approach from models other than those from *Pseudomonas putida*. For any discrepancy between GPR associations from CarveMe and those from BBH approach, we checked IMG and/or KEGG and/or iPau1129 model to assign correct gene associations. Any reactions that did not have associated GPRs or with no evidence to be present in PA14 were discarded in this process. We also removed unnecessary reactions that lead to cyclic production and consumption of metabolites during this process. The list of deleted reactions is provided (Supplementary Data 1). We iteratively improved and validated the model using substrate utilization[2] and gene essentiality[5] data which led to the modification of the biomass reaction and more changes to GPR associations. We also added any genes from iPau1129 that were not associated with the model at that time.

2. Reaction Mass and Charge Balance

For mass-and-charge balance, metabolite formula and charge were assigned using *i*JN1462 first, and then *i*ML1515. Next, we manually checked the remaining metabolites on multiple databases including BiGG[6], MetaNetX[9], PubChem[10] or ModelSEED[11] to identify the correct formula and charge. Finally, if the information for metabolites could not be found in the aforementioned databases, we assigned the formula and charge by balancing the reactions that contain only those metabolites as the sole undetermined ones.

3. Manual Reconstruction

Reactions related to anaerobic metabolism, phenazine-associated metabolism, terminal oxidases and alternative terminal oxidases, thiamine metabolism, nucleotide metabolism, n-alkane metabolism, oxalate production, anaerobic quinone production, rubredoxin-based metabolism, reactive oxygen species (ROS), and H+-translocating NADH:quinone oxidoreductases were added using annotation in KEGG and/or extensive knowledge from literature. All the added reactions are listed (Supplementary Data 2).

4. Medium formulation

Six different media were used in the study. LB and SCFM composition and uptake bounds were provided by Papin Lab. Likewise, a minimal medium composition was also generously provided by Papin Lab. By default, these media and their respective flux constraints were used unless stated otherwise. For case studies, the minimal medium composition derived from the respective studies was used[13, 14, 15]. The fluxes of minimal medium provided by the Papin lab were used as constraints on these three minimal media.

1. M9 (Current Protocol): The medium was derived from Current Protocols in Molecular Biology[13] which was provided by Brad Poulsen from Eric Lander's lab. This medium was used for core essential genes assessment study and gluconate production study.
2. MOPS (LaBauve): The medium was derived from another study[14]. The medium was used for MFA flux comparisons.

3. M9 (Meylan): The medium was derived from Meylan *et al.*[15].

In the minimal medium, ferrous ion (Fe^{2+}) was added because the model cannot simulate growth without the ion. For further details on the medium and the uptake rates used, please see Supplementary Data 5.

5. Substrate Utilization Screening and Gene Knockout Study

For the initial carbon source catabolic activity and gene essentiality validation, the same datasets used in Bartell et al.[2] were utilized. For substrate utilization, we simulated *i*SD1509 in minimal medium (Supplementary Table 1) containing individual substrates and optimized for maximum biomass production. Since ModelSEED identifiers were used in Bartell et al.[2], some of the substrate identifiers could not be converted to the BiGG ones, and hence were removed from this analysis. Overall, 87 compounds were compared. The media composition was provided generously by the Papin lab. For gene essentiality comparison, we simulated the model in LB rich medium.

For substrate utilization screening and gene essentiality assessment of *i*SD1509, data collected from separate experimental studies were applied. For carbon source assay, the dataset by Dunphy et al.[16] containing 190 carbon sources was collected. In the collected dataset, since only general metabolite names were provided, the metabolites whose BiGG identifiers could be determined with high confidence were utilized for prediction. Furthermore, since the two experimental datasets (ref. 2 and ref. [16]) showed discrepancy in *P. aeruginosa*'s ability to grow on eight substrates, those compounds were removed from the analysis. Hence, the model was simulated on minimal medium containing 123 individual substrates for comparison. For

assessing the gene essentiality, we used the dataset from a recent study by Poulsen *et al.*[17]. In this dataset, core essential genes were defined as essential genes in five media conditions and nine different strains. We analyzed these core essential genes in LB medium, SCFM (Synthetic Cystic Fibrosis Medium) and glucose minimal medium. For gene essentiality comparison in glucose minimal medium, iron had to be added even though the media used in the study[17] presumably did not contain iron.

The metrics for the comparison of model predictions and experimental data are defined as follows:

$$Precision = \frac{TP}{TP + FP}$$

$$Negative\ predictive\ accuracy = \frac{TN}{TN + FN}$$

$$Recall = \frac{TP}{EP}$$

$$Specificity = \frac{TN}{EN}$$

$$Accuracy = \frac{TP + TN}{EP + EN}$$

$$MCC = \frac{TP * TN - FP * FN}{\sqrt{(TP + FP)(TP + FN)(TN + FP)(TN + FN)}}$$

where, TP: true positive, FN: false negative, FP: false positive, TN: true negative, EP: experimental essential genes, and EN: experimental non-essential genes.

6. Sensitivity analysis for pyocyanin production rate computation

Oxygen uptake rate was gradually increased from 0 $mmol.gDW^{-1}\ hr^{-1}$ to 20 $mmol.gDW^{-1}\ hr^{-1}$ in step 0.01 $mmol.gDW^{-1}\ hr^{-1}$. The pyocyanin synthesis flux was computed for this range to determine the range of oxygen uptake rates to use for future simulations.

7. Constraints and objective functions for simulations

Unless otherwise stated, the simulations were carried out using the following objective function and set of constraints.

$$\begin{aligned} \max \quad & Z \\ s.t. \quad & Sv = 0 \\ & l \leq v \leq u \end{aligned}$$

where Z = biomass production
S = Stoichiometric matrix
v = Reaction flux
l = Lower flux bound
u = Upper flux bound

Ubiquinone Flux-sum

For this case, select reaction fluxes are constrained.

$$v_{off} = 0 \ \forall \ v \in R_{select}$$

$$v_{condition} \geq L$$

$$v = 0 \ \mathit{if} \ \ v \in R_{gene}$$

where R_{select} = Reactions associated with UQ8 & exchange reaction for either oxygen or nitrate depending on the condition (e.g., for aerobic condition, nitrate exchange is shut down). In anaerobic conditions, all the reactions related to oxygen-associated terminal oxidases are turned off.

$v_{condition}$ = Reactions associated with the condition being simulated in (i.e., aerobic or anaerobic).

L = Lower bound for the reaction.

R_{gene} = Reaction catalyzed by the knockout gene if and only if reaction not catalyzed by isozyme.

Pyocyanin production

For this case, the oxygen uptake rate and pyocyanin synthesis production flux are “fixed” for a wide range as described in the main text.

$$v = \text{Fixed } if\ v \in R_{o2}, R_{pyosyn}$$

where R_{o2} = Oxygen uptake reaction

R_{pyosyn} = *Pyocyanin synthesis reaction*

Gluconate overproduction

For various mutants, the gluconate yield (gluconate production flux normalized glucose uptake flux) is simulated by constraining the reactions catalyzed by the enzymes encoded by certain genes. In this case, for any reaction containing both UQ9 and UQ8 as cofactors, the yield was averaged.

$$v = 0\ if\ \ v \in R_{gene}$$

where R_{gene} = Reaction catalyzed by the knockout gene if and only if reaction not catalyzed by isozyme.

For the FVA analysis of the wildtype and *edd* mutant, the formulation is:

$$\begin{aligned} & max, min \quad v_i \\ \text{s.t.} \quad & Sv = 0 \\ & c^T v = Z \\ & 0 \le v_i \le u_i \end{aligned}$$

where v_i = select i reactions

c = Objective function vector
u_i = upper flux bound for select i reactions

Metabolite supplementation increasing proton motive force activity

For the flux sampling analysis, no objective function was used. The constraint applied is:

$$v_z \geq 0.9 * v_{\mathrm{fba}}$$

where v_z = Biomass reaction flux for sampling
v_{fba} = Biomass reaction flux computed using FBA

Supplementary Figures

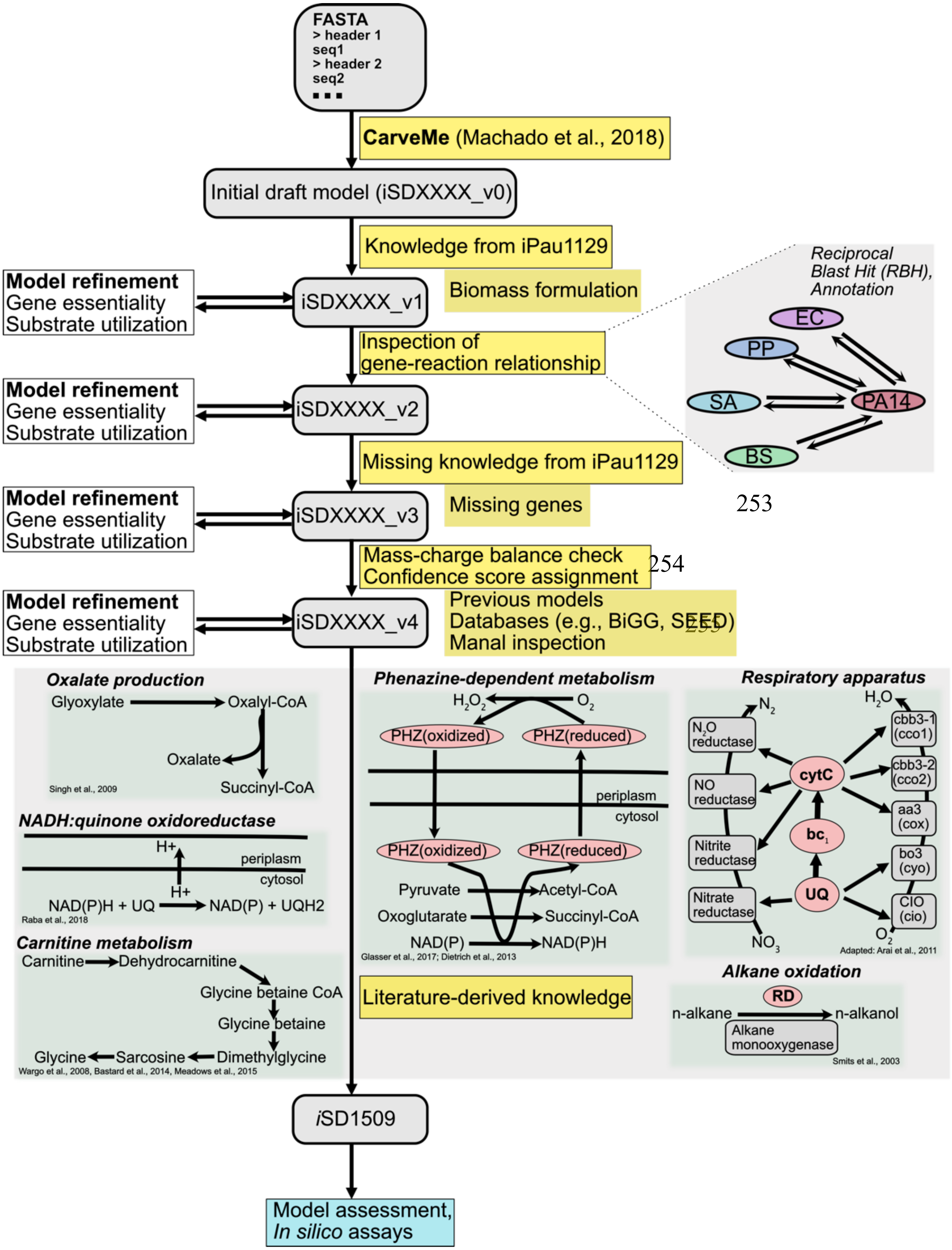

Supplementary Figure 1. The working pipeline for building the genome-scale reconstruction of *P. aeruginosa* PA14. Both automated and semi-automated methods were applied in this pipeline, and the reconstruction was gap-filled using iPau1129 model wherever necessary. Furthermore, strain-specific reactions were added after extensive literature curation. At each stage, the model was validated and improved using gene essentiality and substrate utilization data from[2].

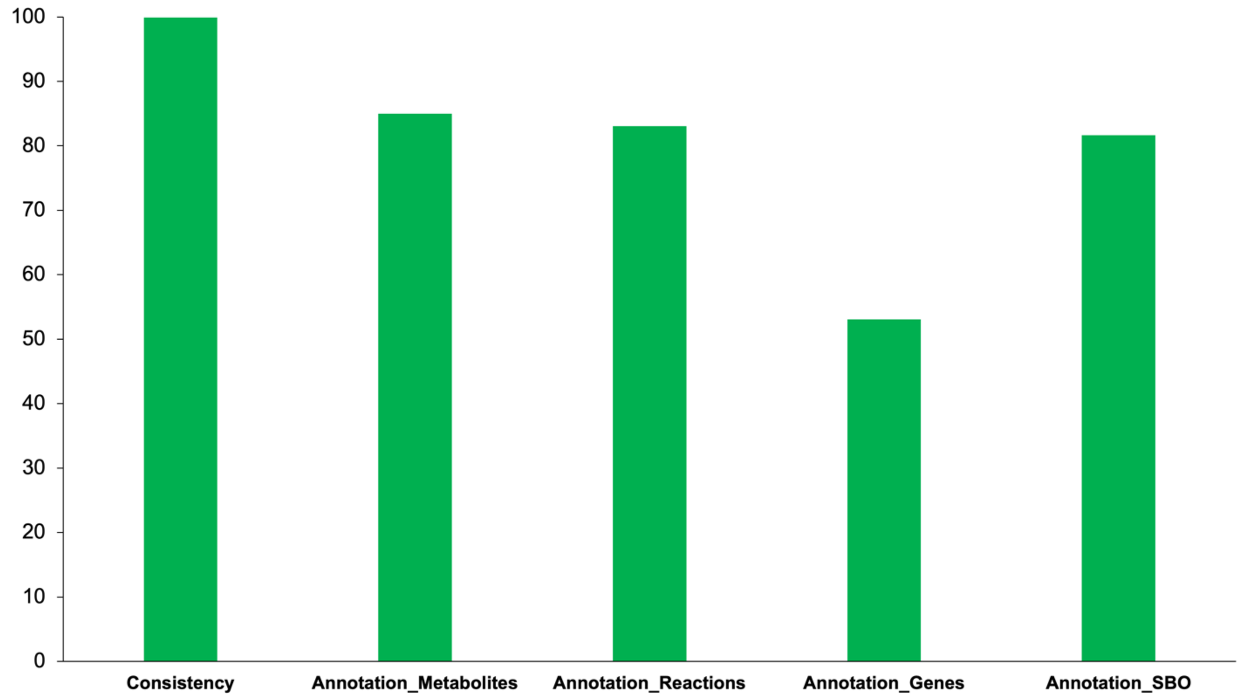

Supplementary Figure 2. Memote score for *i*SD1509. Overall, the score for the model is 88%. Please find the Memote report in the Supplementary Data 6.

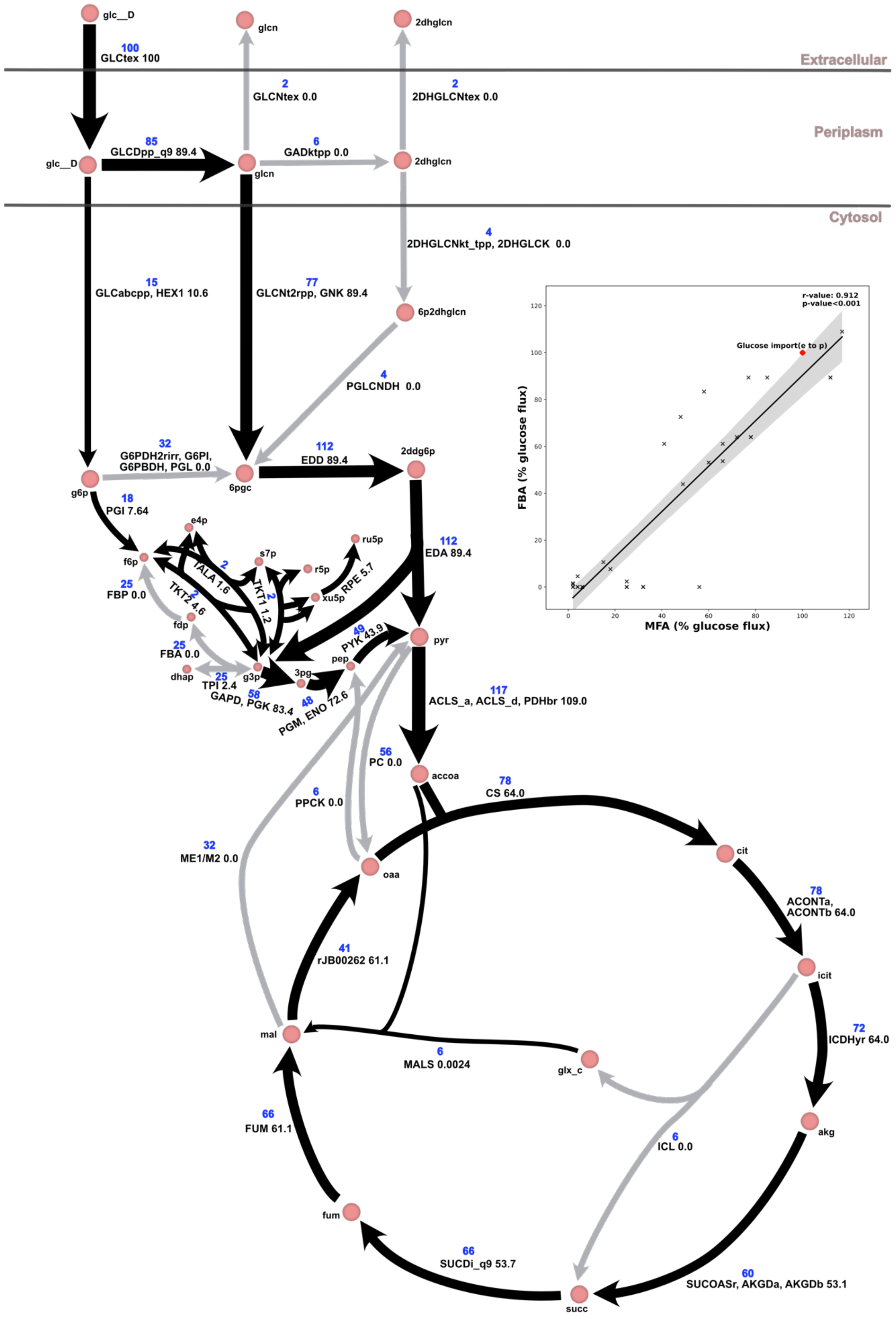

Extracellular
Periplasm
Cytosol
glc__D
glcn
2dhglcn
100
GLCtex 100
2
GLCNtex 0.0
2
2DHGLCNtex 0.0
85
GLCDpp_q9 89.4
6
GADktpp 0.0
glc__D
glcn
2dhglcn
4
2DHGLCNkt_tpp, 2DHGLCK 0.0
15
GLCabcpp, HEX1 10.6
77
GLCNt2rpp, GNK 89.4
6p2dhglcn
4
PGLCNDH 0.0
32
G6PDH2rirr, G6PI,
G6PBDH, PGL 0.0
112
EDD 89.4
2ddg6p
g6p
6pgc
18
PGI 7.64
e4p
ru5p
s7p
r5p
f6p
2
TALA 1.6
2
TKT1 1.2
RPE 5.7
xu5p
112
EDA 89.4
25
FBP 0.0
2
TKT2 4.6
fdp
49
PYK 43.9
pyr
25
FBA 0.0
pep
dhap
g3p
3pg
25
TPI 2.4
58
GAPD, PGK 83.4
48
PGM, ENO 72.6
117
ACLS_a, ACLS_d, PDHbr 109.0
56
PC 0.0
accoa
6
PPCK 0.0
78
CS 64.0
32
ME1/M2 0.0
cit
oaa
78
ACONTa,
ACONTb 64.0
41
rJB00262 61.1
icit
72
ICDHyr 64.0
mal
6
MALS 0.0024
glx_c
66
FUM 61.1
6
ICL 0.0
akg
fum
66
SUCDi_q9 53.7
60
SUCOASr, AKGDa, AKGDb 53.1
succ
r-value: 0.912
p-value<0.001
Glucose import(e to p)
FBA (% glucose flux)
MFA (% glucose flux)
0
20
40
60
80
100
120

Supplementary Figure 3. FBA simulations agree with MFA experimental data[18]. In the figure, the width of arrows represents the percentage of glucose flux. Only absolute values are shown here. All the zero value arrows are made grey for clarity. The MFA average values are written in the blue font if their respective reactions were found in the reconstruction. Lumped reactions are represented by multiple reactions separated by commas. Since two possible reactions (ME1 and ME2) are present in the M-model for the reactions catalyzed by the malic enzyme, both are shown separated by ”/”. The box inset is a correlation plot that corroborates the fact that the majority of the predictions agree with the MFA results (correlation coefficient of 0.91 ($p<0.001$)). Grey bands denote 95% confidence interval. Within the plot, glucose import (from extracellular to periplasm) has been indicated for reference and marked with a red diamond.

A.

Comparing gene essentiality using data from Liberati et al.

	***i*SD1509**	**iPau1129**
**Precision**	47.2	49.1
**Negative Predictive Accuracy**	96.3	95.8
**Recall**	62.1	56.8
**Specificity**	93.3	94.3
**Accuracy**	90.6	91.1
**MCC**	0.49	0.48

B.

		***i*SD1509 prediction**	
		**+**	**-**
**Experimental observation**	**+**	TP 63	FN 57
	**-**	*FP* 70	*TN* 1319

**Precision**	**Negative Predictive Accuracy**	**Recall**	**Specificity**	**Accuracy**	**MCC**
47.4	95.9	52.5	95.0	91.6	0.45

Supplementary Figure 4. Comparison of gene essentiality between *i*SD1509 and iPau1129. A) Comparing shared genes (n=1,085) between the two models for dataset retrieved from Liberati *et al.*[5]. B) Computation of gene essentiality for all the genes present in *i*SD1509 using the same dataset[5].

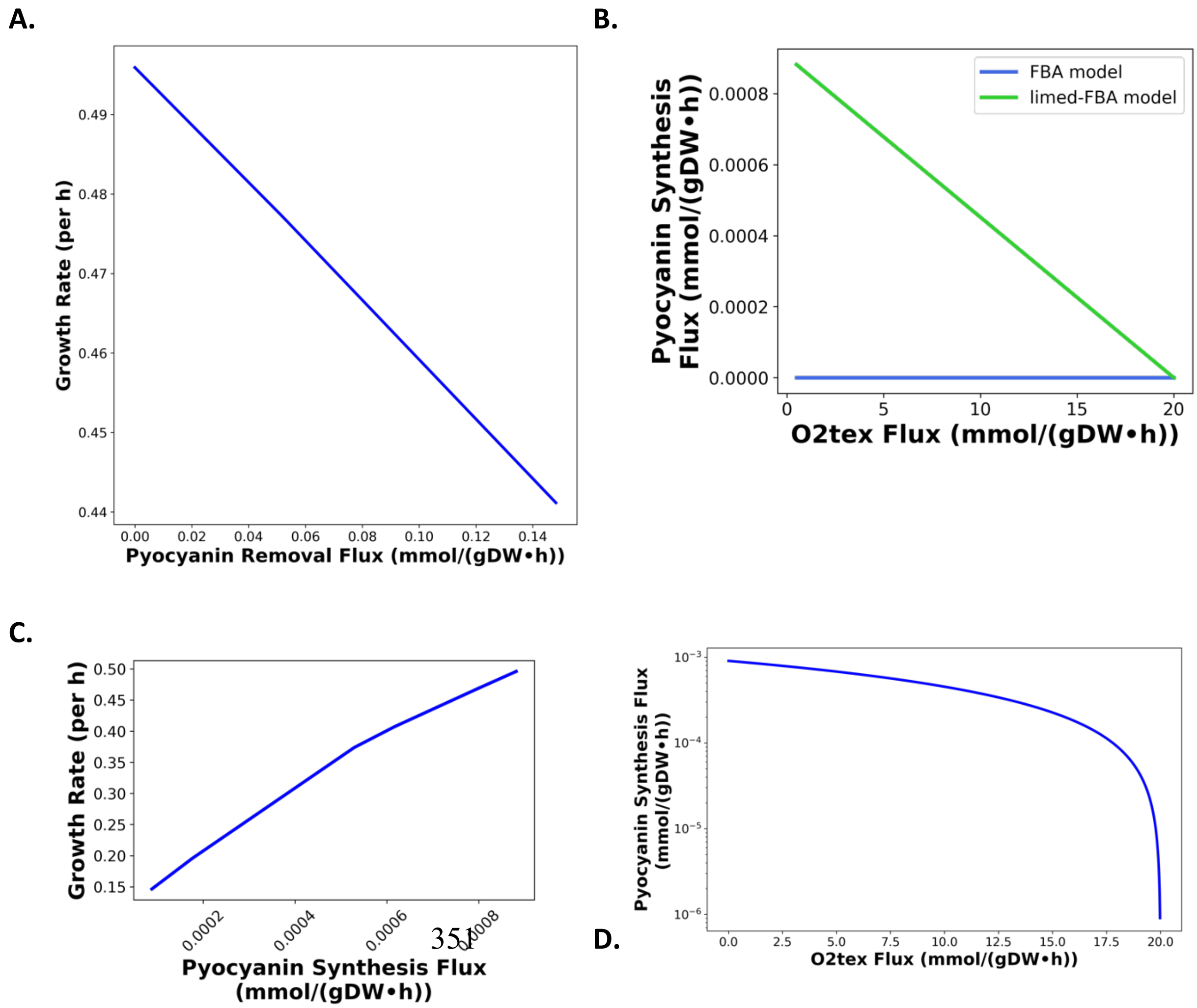

Supplementary Figure 5. Using *i*SD1509_limed to demonstrate that the phenazine production is crucial for the pathogen survival. A) A simple formulation of pyocyanin removal (i.e., a demand reaction) was added to the model, and the simulations were performed in oxygen-limited condition (O2tex (oxygen transport from extracellular to periplasm) upper bound: 0.5 mmol gDW^{-1} hr^{-1}). As the pyocyanin removal flux increased, the model predicted that the biomass production of *P. aeruginosa* also lowered. Please note that this simulation can also be performed using the FBA model. B) Demonstration of the application of limed-FBA that predicted phenazine production is higher at lower oxygen availability condition. Since pyocyanin is not part of the biomass reaction in *i*SD1509, it does not dilute as the model

simulates growth. Hence, the FBA model (blue) cannot predict the synthesis of pyocyanin. C) In oxygen-limited condition (O2tex upper bound: 0.5 mmol gDW^{-1} hr^{-1}), the biosynthesis of phenazine can affect the growth of the pathogen considerably. D) Sensitivity analysis for pyocyanin synthesis flux and oxygen uptake rate. Pyocyanin production slowly decreases over a wide range, but then drastically drops near 20 mmol gDW^{-1} hr^{-1}. Using this result, we chose 0.5 mmol gDW^{-1} hr^{-1} - 10 mmol gDW^{-1} hr^{-1} as the oxygen uptake range to further investigate the effect of oxygen uptake rate and pyocyanin synthesis on the biomass production of *P. aeruginosa*.

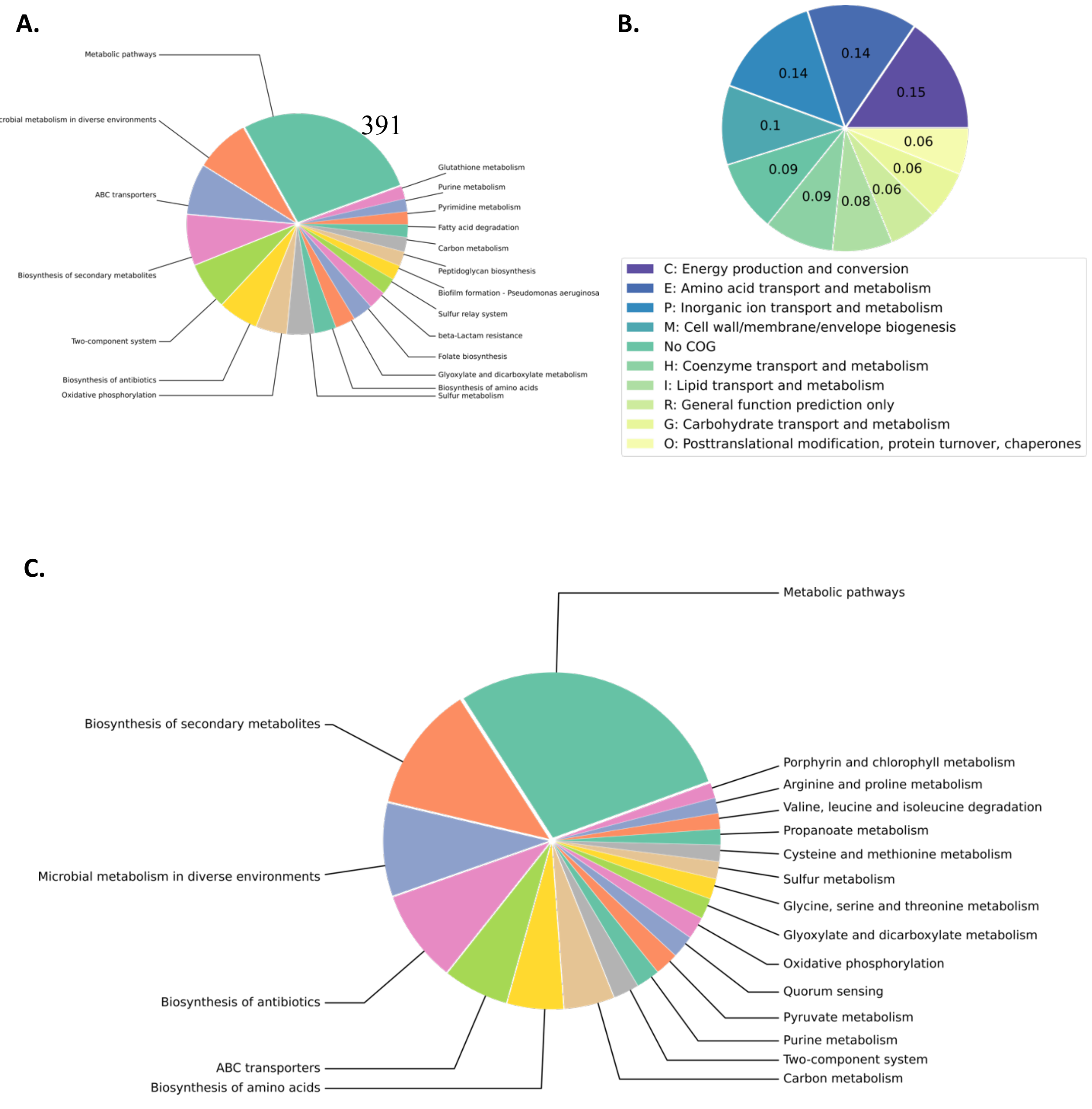

Supplementary Figure 6. Pathway enrichment of the genes present in *i*SD1509. A) The top 20 KEGG pathways of unique genes in *i*SD1509. B) The top 20 COG categories of unique genes in *i*SD1509. C) The top 20 KEGG pathways of all the genes present in *i*SD1509.

Supplementary Table 1. Minimal media compositions. For more details, please refer to Supplementary Data 5.

Model Assessment and Gluconate Prediction[a]	Drug Potentiation/Protection[b]
H_2O	H_2O
PO_4^{3-}	PO_4^{3-}
CO_2	CO_2
Fe^{2+}	Fe^{2+}
SO_4^{2-}	SO_4^{2-}
H^+	H^+
K^+	K^+
Na^+	Na^+
NH_4^+	NH_4^+
O_2	O_2
Mg^{2+}	Mg^{2+}
	Citrate

[a] For model assessment and gluconate prediction study, glucose was used as the carbon substrate.

[b] For the drug potentiation/protection investigation, either fumarate or glyoxylate were used as carbon substrates.

Supplementary Table 2. Strains and the models used for the determination of gene-protein-reaction (GPR) associations.

Priority	Strain Name	Genome ID	ANI1->2[a]	Models used
**1**	*Pseudomonas putida* KT2440	637000222	77.759	iJN1462, iJN746
**2**	*Escherichia coli* UTI89	637000109	67.768	iUTI89_1310
**3**	*Escherichia coli* str. K-12 substr. MG1655	646311926	67.712	iML1515, iJR904, iAF1260b
**4**	*Escherichia coli* str. K-12 substr. W3110	637000110	67.702	iY75_1357
**5**	*Escherichia coli* ED1a	643348545	67.698	iECED1_1282
**6**	*Escherichia coli* KO11FL	2513237200	67.675	iEKO11_1354
**7**	*Geobacter metallireducens* GS-15	637000119	66.702	iAF987
**8**	*Mycobacterium tuberculosis* H37Rv	637000173	66.467	iEK1008, iNJ661
**9**	*Synechocystis* sp. PCC 6803	637000315	62.137	iSynCJ816
**10**	*Bacillus subtilis* subtilis 168	646311909	60.516	iYO844
**11**	*Clostridioides difficile* 630	640069308	59.551	iCN900
**12**	*Clostridium ljungdahlii* DSM 13528	648028017	56.670	iHN637
**13**	*Staphylococcus aureus* subsp. aureus USA300_TCH1516	643692036	56.027	iYS854

[a] The strains are prioritized by their Average Nucleotide Identity (ANI) score (from strain 1 (*P. aeruginosa* PA14) to strain 2 (other strains)) as computed in the JGI server.

Supplementary Table 3. Biomass composition for aerobic and anaerobic conditions.

Metabolite ID	Metabolite Name
datp	DATP
dctp	DCTP
dgtp	DGTP
dttp	DTTP
ctp	CTP
gtp	GTP
utp	UTP
ala__L	L-Alanine
arg__L	L-Arginine
asn__L	L-Asparagine
asp__L	L-Aspartate
cys__L	L-Cysteine
gln__L	L-Glutamine
glu__L	L-Glutamate
gly	Glycine
his__L	L-Histidine
ile__L	L-Isoleucine
leu__L	L-Leucine
lys__L	L-Lysine
met__L	L-Methionine
phe__L	L-Phenylalanine
pro__L	L-Proline
ser__L	L-Serine
thr__L	L-Threonine
trp__L	L-Tryptophan
tyr__L	L-Tyrosine
val__L	L-Valine
glycogen	Glycogen
pe160	Phosphatidylethanolamine (dihexadecanoyl, n-C16:0)
pe180	Phosphatidylethanolamine (dioctadecanoyl, n-C18:0)
pe161	Phosphatidylethanolamine (dihexadec-9enoyl, n-C16:1)
pe181	Phosphatidylethanolamine (dioctadec-11-enoyl, n-C18:1)
pe_pa_17_0_cyc	pe170cyc[a]
pe_pa_19_0_cyc	pe190cyc[a]
pg160	Phosphatidylglycerol (dihexadecanoyl, n-C16:0)
pg180	Phosphatidylglycerol (dioctadecanoyl, n-C18:0)
pg161	Phosphatidylglycerol (dihexadec-9-enoyl, n-C16:1)
pg181	Phosphatidylglycerol (dioctadec-11-enoyl, n-C18:1)

pg_pa_17_0_cyc	pg170cyc[a]
pg_pa_19_0_cyc	pg190cyc[a]
clpn160	Cardiolipin (tetrahexadecanoyl, n-C16:0)
clpn180	Cardiolipin (tetraoctadecanoyl, n-C18:0)
clpn161	Cardiolipin (tetrahexadec-9-enoyl, n-C16:1)
clpn181	Cardiolipin (tetraoctadec-11-enoyl, n-C18:1)
clpn_pa_17_0_cyc	clpn170cyc[a]
clpn_pa_19_0_cyc	clpn190cyc[a]
peptido_pa	Peptidoglycan polymer (n subunits)
pa_core_lipida	Pseudomonas aeruginosa LPS core + KDO2-lipidA
ptrc	Putrescine
q9h2	Ubiquinol-9
mlthf	5,10-Methylenetetrahydrofolate
coa	Coenzyme A
2fe2s	[2Fe-2S] iron-sulfur cluster
atp	ATP
h2o	H2O
h	H+
ppi	Diphosphate
adp	ADP
pi	Phosphate
thmpp	Thiamine diphosphate

*The aerobic biomass reaction is provided. The composition was derived from Bartell *et al.*[2] For anaerobic biomass reaction, *thmpp* (highlighted in orange) is removed.

[a]The metabolite names were directly taken from the study[2].

Supplementary References

1. Machado D, Andrejev S, Tramontano M, Patil KR. Fast automated reconstruction of genome-scale metabolic models for microbial species and communities. *Nucleic Acids Res* **46**, 7542-7553 (2018).

2. Bartell JA*, et al.* Reconstruction of the metabolic network of Pseudomonas aeruginosa to interrogate virulence factor synthesis. *Nat Commun* **8**, 14631 (2017).

3. Chen I-MA*, et al.* The IMG/M data management and analysis system v. 6.0: new tools and advanced capabilities. *Nucleic Acids Research* **49**, D751-D763 (2021).

4. Kanehisa M, Furumichi M, Sato Y, Ishiguro-Watanabe M, Tanabe M. KEGG: integrating viruses and cellular organisms. *Nucleic Acids Research* **49**, D545-D551 (2021).

5. Liberati NT*, et al.* An ordered, nonredundant library of Pseudomonas aeruginosa strain PA14 transposon insertion mutants. *Proceedings of the National Academy of Sciences* **103**, 2833-2838 (2006).

6. King ZA*, et al.* BiGG Models: A platform for integrating, standardizing and sharing genome-scale models. *Nucleic acids research* **44**, D515-D522 (2016).

7. Nogales J*, et al.* High-quality genome-scale metabolic modelling of Pseudomonas putida highlights its broad metabolic capabilities. *Environ Microbiol* **22**, 255-269 (2020).

8. Monk JM*, et al.* i ML1515, a knowledgebase that computes Escherichia coli traits. *Nature biotechnology* **35**, 904-908 (2017).

9. Moretti S, Tran Van Du T, Mehl F, Ibberson M, Pagni M. MetaNetX/MNXref: unified namespace for metabolites and biochemical reactions in the context of metabolic models. *Nucleic Acids Research* **49**, D570-D574 (2020).

10. Kim S*, et al.* PubChem in 2021: new data content and improved web interfaces. *Nucleic Acids Research* **49**, D1388-D1395 (2020).

11. Seaver SM*, et al.* The ModelSEED Biochemistry Database for the integration of metabolic annotations and the reconstruction, comparison and analysis of metabolic models for plants, fungi and microbes. *Nucleic acids research* **49**, D575-D588 (2021).

12. Galperin MY, Wolf YI, Makarova KS, Vera Alvarez R, Landsman D, Koonin EV. COG database update: focus on microbial diversity, model organisms, and widespread pathogens. *Nucleic Acids Research* **49**, D274-D281 (2020).

13. Evans A, Ausubel FM. *Current protocols in molecular biology*. Greene Pub. Associates and Wiley-Interscience (1987).

14. LaBauve AE, Wargo MJ. Growth and laboratory maintenance of Pseudomonas aeruginosa. *Current protocols in microbiology* **25**, 6E. 1.1-6E. 1.8 (2012).

15. Meylan S*, et al.* Carbon sources tune antibiotic susceptibility in Pseudomonas aeruginosa via tricarboxylic acid cycle control. *Cell chemical biology* **24**, 195-206 (2017).

16. Dunphy LJ, Yen P, Papin JA. Integrated Experimental and Computational Analyses Reveal Differential Metabolic Functionality in Antibiotic-Resistant Pseudomonas aeruginosa. *Cell Syst* **8**, 3-14 e13 (2019).

17. Poulsen BE*, et al.* Defining the core essential genome of Pseudomonas aeruginosa. *Proc Natl Acad Sci U S A* **116**, 10072-10080 (2019).

18. Kohlstedt M, Wittmann C. GC-MS-based (13)C metabolic flux analysis resolves the parallel and cyclic glucose metabolism of Pseudomonas putida KT2440 and Pseudomonas aeruginosa PAO1. *Metab Eng* **54**, 35-53 (2019).
