# Supplementary material for: Genome-scale model of Pseudomonas aeruginosa metabolism unveils virulence and drug potentiation
Source: Commun Biol. 2023 Feb 10;6:165. doi: 10.1038/s42003-023-04540-8 (PMC9918512; doi:10.1038/s42003-023-04540-8)
Supplement: Supplementary file 2 — Description of Additional Supplementary Data [file 42003_2023_4540_MOESM2_ESM.docx]

**Description of Additional Supplementary Files**

**File name:** Supplementary Data 1

**Description:** The list of deleted reactions from the draft model.

**File name:** Supplementary Data 2

**Description:** All the added reactions to the draft model.

**File name:** Supplementary Data 3

**Description:** Genes from iPau1129 that are missing in iSD1509.

**File name:** Supplementary Data 4

**Description:** Metabolites (with descriptions) that required manual curation.

**File name:** Supplementary Data 5

**Description:** Medium and uptake rates used in this study.

**File name:** Supplementary Data 6

**Description:** Memote Report for iSD1509.

**File name:** Supplementary Data 7

**Description:** The source data underlying Figs. 2, 3, 4, and 5.
